# Gene expression profiling of whole blood: A comparative assessment of RNA-stabilizing collection methods

**DOI:** 10.1371/journal.pone.0223065

**Published:** 2019-10-10

**Authors:** Duncan E. Donohue, Aarti Gautam, Stacy-Ann Miller, Seshamalini Srinivasan, Duna Abu-Amara, Ross Campbell, Charles R. Marmar, Rasha Hammamieh, Marti Jett

**Affiliations:** 1 Integrative Systems Biology Program, U.S. Army Center for Environmental Health Research, Fort Detrick, MD, United States of America; 2 The Geneva Foundation, Fort Detrick, MD, United States of America; 3 Steven and Alexandra Cohen Veterans Center for the Study of Posttraumatic Stress and Traumatic Brain Injury, Department of Psychiatry, NYU School of Medicine, New York, NY, United States of America; 4 Advanced Biomedical Computing Center, Frederick, MD, United States of America; University of Surrey, UNITED KINGDOM

## Abstract

Peripheral Blood gene expression is widely used in the discovery of biomarkers and development of therapeutics. Recently, a spate of commercial blood collection and preservation systems have been introduced with proprietary variations that may differentially impact the transcriptomic profiles. Comparative analysis of these collection platforms will help optimize protocols to detect, identify, and reproducibly validate true biological variance among subjects. In the current study, we tested two recently introduced whole blood collection methods, RNAgard^®^ and PAXgene^®^ RNA, in addition to the traditional method of peripheral blood mononuclear cells (PBMCs) separated from whole blood and preserved in Trizol reagent. Study results revealed striking differences in the transcriptomic profiles from the three different methods that imply *ex vivo* changes in gene expression occurred during the blood collection, preservation, and mRNA extraction processes. When comparing the ability of the three preservation methods to accurately capture individuals’ expression differences, RNAgard^®^ outperformed PAXgene^®^ RNA, and both showed better individual separation of transcriptomic profiles than PBMCs. Hence, our study recommends using a single blood collection platform, and strongly cautions against combining methods during the course of a defined study.

## Introduction

Peripheral blood remains a popular tissue to interrogate biological state, mainly due to its availability and minimally invasive mode of collection. Also, its systemic connectivity to numerous bodily tissues renders it a valuable and rich source for detection and identification of human biomarkers. These blood biomarkers can be used extensively in a wide range of applications from diagnostics, such as monitoring disease progression to therapeutics, drug development, and assessing patient responses to medical treatment. While mRNA quantification provides a powerful functional readout which bridges DNA sequence and protein levels, maintaining RNA integrity of the peripheral blood during its collection and preservation prior to analysis poses numerous technical challenges.

Post-collection, the nucleic acids in blood are susceptible to oxidative damage and nuclease attack [1]. Additionally, the relatively unstable intracellular RNAs are subjected to both transcript induction [2] and transcript degradation[1, 3], resulting in altered gene expression *ex vivo*. Together, these experimental variations complicate the detection of true biological variance. Various stages after phlebotomy, including blood collection, transportation and storage, along with processing steps such as RNA isolation method and choice of microarray platform, can influence gene expression profiles [4]. Hence, optimization of these technical variables is necessary to uncover the true biological patterns inherent in blood. Although novel commercial systems for collection and rapid stabilization of peripheral blood RNA are frequently added to the inventory, systematic research updates demonstrating the impact of these solutions on whole genome profiling is inadequate. In an effort to bridge this knowledge gap, we compared transcriptomic profiles generated using two commercial formulations, namely PAXgene^®^RNA Blood RNA tube (PreAnalytiX, Qiagen BD, Valencia, CA) and RNAgard^^®^^ Blood Tube (Biomatrica, Inc., San Diego, CA). Both of these RNA collection systems use proprietary reagents that lyse blood cells immediately after collection in the tubes, inhibit RNA degradation, and block induction of new transcripts [5, 6]. Multiple technical replicates were compared to address the issues related to the reproducibility of the results utilizing collection tubes of different manufacturers.

Prior studies comparing PAXgene^®^RNA tubes to Tempus^™^ Blood RNA Tubes (Applied Biosystems) show that the choice of collection tubes can critically impact differential expression patterns. According to Asare *et al*., the Tempus^™^ system preserved the *in-vivo* transcription profiles better, thus leading to the identification of a greater number of gene expression changes and reflecting the true biology of specific sets of inducible genes [7]. In another study, Menke *et al*. identified a mere 54% overlap in the glucocorticoid receptor-stimulated gene expression profiles between these two commonly used commercial systems [8]. They reported that only differentially regulated transcripts with large, robust, and significant fold changes are detected in both PAXgene^®^RNA and Tempus^™^ tubes. Recently, Nikula and colleagues corroborated these findings; they noted hundreds of transcripts associated with many blood immune cell functions and canonical pathways were differentially expressed because of technical bias between PAXgene^®^RNA and Tempus^™^ RNA preservation methods [9]. Conversely, research conducted by Häntzsch *et al*. claimed that the choice between the PAXgene^®^RNA and Tempus^™^ RNA collection tubes does not affect mRNA expression profiling, but does alter miRNA levels [10]. More recently, Meyer *et al*. observed significant differences between these two methods during their attempt to optimize a combination of the blood collection system and RNA extraction procedure to generate reproducible gene expression results from human blood samples [11].

In addition to the PAXgene^®^RNA and Tempus^™^ Blood RNA systems studied in these reports, RNAgard^^®^^ Blood Tubes is another commercially available blood collection system designed for rapid stabilization to yield RNA suitable for genome-wide expression profiling. RNAgard^®^, however, remains largely unexplored and, to the best of our knowledge, the utility of RNAgard^®^ has yet to be systematically compared to any other method. Hence, in this investigation, we compared genome-wide expression profiles obtained from two different whole blood RNA collection systems: PAXgene^®^RNA and RNAgard^®^. For reference purposes, peripheral blood mononuclear cells (PBMCs) preserved in Trizol have been included as an additional RNA preservation system. Although RBC-depletion from blood samples has been shown to improve preserving the quality of RNA in Trizol [12], processing delays as little as 4 hours can affect PMBC gene expression profiles, especially in genes related to immune response [12, 13]. Thus, our investigation is aimed at broadening awareness among researchers of the biases introduced due to differences in blood collection methods, and possibly better determine an appropriate system for clinical studies. In this report, we present the relative merits and the inherent biases of the RNAgard^®^ collection system in comparison with the widely used PAXgene^®^RNA, and the conventional method of PBMCs preserved in TRIzol.

## Materials and methods

### Participant selection

This study comprises active duty Army personnel of the 101st Airborne at Fort Campbell, Kentucky, assessed before and after being deployment. The first phase of recruitment occurred during a two-week period immediately prior to deployment with a follow-up phase 2 and 3 after deployment. This study used samples from phase 1 sample collection. The study was conducted in accord with ethical principles for the conduct of human research as specified in the latest version of the Declaration of Helsinki.The study was approved by the Institutional Review Board of NYU School of Medicine, Human Research Protection Office of the United States Army at Ft. Detrick MD and Army Command of the 101st Airborne at Ft. Campbell Kentucky.

### Blood collection and nucleic acid isolation

Peripheral blood was collected from eight healthy volunteers using a 21-gauge butterfly needle and catheter after obtaining informed consent. For each subject, four samples of blood were collected and dispensed in into a PAXgene^®^RNA tube (2.5ml whole blood) (PreAnalytiX), an RNAgard^®^ Blood Tube (2.5ml whole blood) (Biomatrica), and duplicate CPT tubes (8.0 ml whole blood in each tube) (Becton Dickinson, Franklin Lakes, NJ). All PAXgene and RNAgard tubes were inverted about 10 times after collection and incubated at room temperature for 2 hours. Tubes were subsequently frozen at -20°C for at least 24 hours followed by storage at -80°C … In parallel, PBMCs separation was carried out using BD Vacutainer^®^ CPT^™^ (BD Biosciences) at the collection site following the manufacturer’s instructions. This was accomplished by centrifugation of the samples at 1700 × G for 20 minutes at room temperature within two hours of blood collection, followed by carefully removing PBMCs using a transfer pipette. The PBMCs were then washed using PBS and the cell pellet was immediately stabilized by adding 500ul of Trizol reagent (Invitrogen, Karlsruhe, Germany). All tubes and sample aliquots were transported from the clinical site on dry ice.

Frozen PAXgene^®^RNA tubes were handled following our basic laboratory protocol based off PreAnalytiX specimen handling and enhance yield procedures from PAXgene Blood RNA MDx Kit Handbook (08/2016). The tubes were thawed overnight at room temperature to ensure complete lysis of blood cells and maximize the mRNA yield. A 2.0 mL (PAXgene^®^RNA + blood) aliquot was used for automated RNA extractions with the PAXgene^®^RNA blood miRNA kit (Qiagen) employing an amended version of the manufacturer’s guidelines on the QIAcube Workstation (Qiagen) [14–16] Briefly, the tubes were centrifuged for 10 min at 3500 g, the supernatant decanted and 1000 μL of RNase-free water added to the pellet. The tube was vortexed to thoroughly re-suspend the pellet, centrifuged for 10 min at 3500 g and the supernatant discarded. The remaining pellet was re-suspended in 350 μL of buffer BM1 by vortexing and transferred onto the QIAcube liquid handler. All subsequent procedures were performed by the Qiacube according to the manufacturer’s recommended protocol. Breifly, The automated RNA purification protocol consists of 2 parts, “PAXgene Blood miRNA Part A” in which the QIAcube performs the steps of the protocol through to elution of RNA in elution buffer, and “PAXgene Blood miRNA Part B” where heat denaturation of samples at 65°C is performed by the QIAcube, with a brief manual intervention between the 2 parts where microcentrifuge tubes, containing the purified RNA, are transferred into the thermoshaker unit of the QIAcube.

Frozen RNAgard^®^ tubes were processed in a similar manner as the PAXgene^®^RNA tubes with a few minor exceptions After thawing the tubes overnight, 4 mL of blood was transferred to a separate tube for RNA extractions. The procedure was adapted after consultation with manufacturer and BioMaxi Precipitation Buffer (Biomatrica) was added to the 4 mL blood in 1:4 ratios. This was followed by incubating the mixture for 15 minutes at room temperature and a quick vortexing was performed. The contents were centrifuged at 4,500 g for 30 minutes, and the supernatant (used for DNA extraction simultaneously) was separated from the pellet. The pellet was used to extract RNA on the Qiacube liquid handler by using the exact same procedure described above for the PAXgene^®^RNA tubes. The quality and quantity of the resulting RNA was measured using the same techniques as for the PAXgene^®^RNA tubes. In order to minimize batch effects, all blood samples were extracted on the same day.

RNA from PBMCs stored in TRIzol was isolated according to the manufacturer's protocol. Briefly, cells were lysed by vortexing in TRIZOL Reagent, which is a monophasic solution of phenol and guanidine isothiocyanate, disrupts the cells and solubilizing cell components. This step is followed by the addition of chloroform which separates the solution into an aqueous phase and an organic phase. The RNA remains in the aqueous phase and is recovered by precipitation with isopropanol. The resulting nucleic acids were quantified using NanoDrop (NanoDrop products, Wilmington, DE, USA). The integrity of the RNA was analyzed using Agilent RNA ScreenTape assay (Agilent Technologies, Inc., CA).

### Global gene expression microarrays

Microarray hybridization and sample labeling were performed according to the Two-Color Microarray-Based Gene Expression Analysis–Low input Quick Amp Labeling- protocol version 6.9 (Agilent Technologies). Agilent RNA Spike-In mix was added to 200 ng of total RNA prior to the labeling reactions to monitor both labeling reactions and microarray performance, following the Two-Color RNA Spike-In Kit protocol (Agilent 5188–5279). Samples were labeled using the Low Input Quick Amp Labeling kit (Agilent 5190–2306) and then hybridized to the Agilent SurePrint G3 Human gene expression v2 8 × 60K Microarray Kit, design ID:039494 (Agilent Technologies, Inc., CA) following the manufacturer’s protocol. Total RNA was reverse transcribed to cDNA, followed by *in vitro* transcription and incorporation of Cy-5 fluorescent dye into the test sample. Universal Human Reference RNA (Stratagene, La Jolla, California) was labeled with Cy-3 fluorescent dye and used as a common reference RNA across all arrays. The samples were purified, dye incorporation and cRNA yield were checked with Nanodrop (NanoDrop products, Wilmington, DE, USA), then simultaneously hybridized to Agilent 8 × 60k slides for 17 hours at 65°C using Agilent’s Gene Expression Hybridaization Kit (Agilent 5188–5442) according to the manufacturer’s instructions. The arrays were washed and scanned according to Agilent protocol using SureScan Microarray Scanner (G2600D, Agilent Technologies, Inc., CA) and two color scan setting for 8x60K array. Microarray performance was assessed by QC metric tool. Following the manufacturer's protocol, Agilent's Stabilization and Drying Solution (Agilent 5185–5979) was used to protect against the ozone-induced degradation of dyes on microarray slides during hybridization and processing steps. The intensity data were extracted using the Feature Extraction 11.5.1.1 software (Agilent Technologies) default parameters. RNA samples obtained from all three collection tube types for each subject were run in duplicates hybridized to separate arrays on a single 8 array slide.

The spike-in RNA demonstrated a linear relation between signal intensity and concentration of the RNA and all datasets were qualified for further analysis. All subsequent analysis was performed using custom R scripts. Median red and green channel intensity values were within array normalized using local polynomial regression, converted to log2 fold differences (M values), and quantile across-array normalized. Duplicate probes were then averaged together. Other common normalization methods were investigated and their use did not change the general findings. Likewise, enforcing a 50% probe variance cutoff filter to highlight technical variation produced very similar results. Gene Ontology (GO) term enrichment analysis was performed using the online bioinformatics resource DAVID [17].

## Results

**Fig 1:**
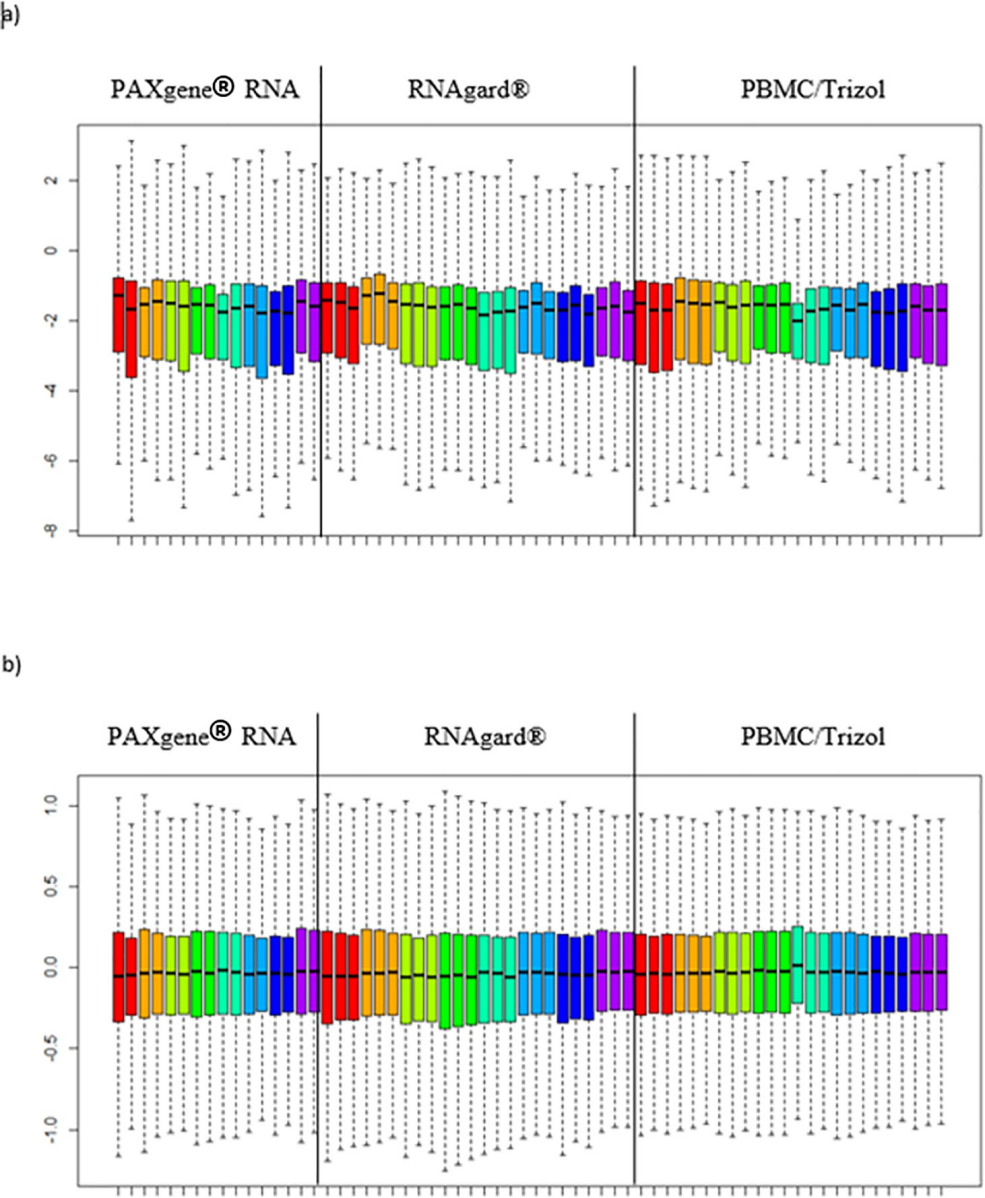
Microarray signal intensity distribuiton before and after normalization. Raw (a) and normalized (b) microarray probe M values obtained for eight different individuals using three different blood collections systems. Colors represent individual donors.

**Fig 2:**
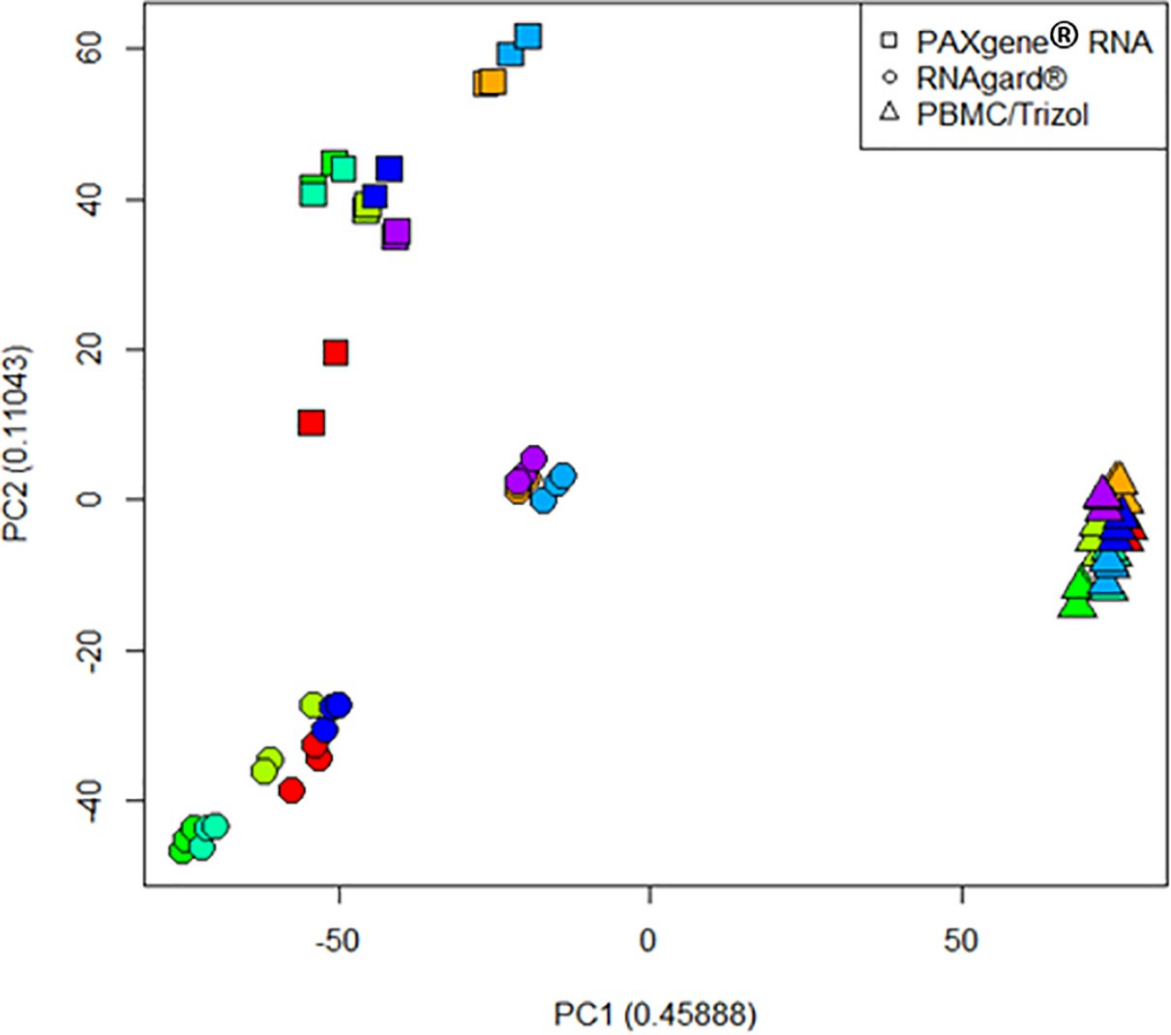
Principal component analysis of technical replicates. Principal Component Analysis (PCA) of normalized mRNA transcript values across individuals (colors) and platforms.

Mean (±SD) TapeScreen RNA integrity number (RIN) values were 8.875 (±0.595) for PBMC/Trizol, significantly higher (two sided unpaired t-test p < 0.0001) than PAXgene^®^RNA at 7.306 (0.595) and RNAgard^®^ at 7.443 (0.614). The PAXgene^®^RNA and the RNAgard^®^ RIN values did not differ significantly from each other. Fig 1 shows raw (a) and within array normalized (b) mRNA expression levels across eight different individuals using three different collection platforms; RNAgard^®^ (triplicate), PAXgene^®^RNA (duplicate), and PBMC/Trizol (triplicate), comprising a total of 64 samples, eight in each set. Overall patterns of expression fold values appear consistent across the three preservation methods. However, as seen in Fig 2, Principal Component Analysis (PCA) revealed considerable differences in mRNA transcript patterns between the RNAgard^®^ and PAXgene^®^RNA preserved samples collected from the same individual using the same microarray platform. As expected based on differing cell type composition, the PBMC samples differed qualitatively from the two whole blood based collection methods. Technical repeat samples (color coded) from individuals tended to cluster together in these two different collection methods; in contrast, PBMCs preserved in Trizol showed less overall variation but poorer separation among individuals (Fig 2). Overall, the profile differences observed between methods were relatively greater than the differences between individual subjects within the methods used.

Relative variability across samples alone does not directly address the ability to accurately quantify biological signal as methods which reduce message abundance variability may do so at the cost of real biological variation. Table 1 shows the relative signal-to-noise (S/N) ratio averaged for technical repeats within and across individual expression correlations using the three different preservation systems. S/N here is defined as the mean ratio of the correlation of within donor probe M values over across donor probe M values for each preservation method. Lower values of S/N signify a reduced ability to differentiate mRNA levels across individuals, whereas relatively higher ratio values imply better quality of technical replicates.

**Table 1:** Comparison of within and across donor probe M value correlations using three different blood collection systems.

	PAXgene^®^RNA	RNAgard^®^	PBMC/Trizol
Mean within donor probe correlation	0.966	0.972	0.968
Mean across donor probe correlation	0.913	0.913	0.927
Ratio	1.058	1.064	1.044

**Fig 3:**
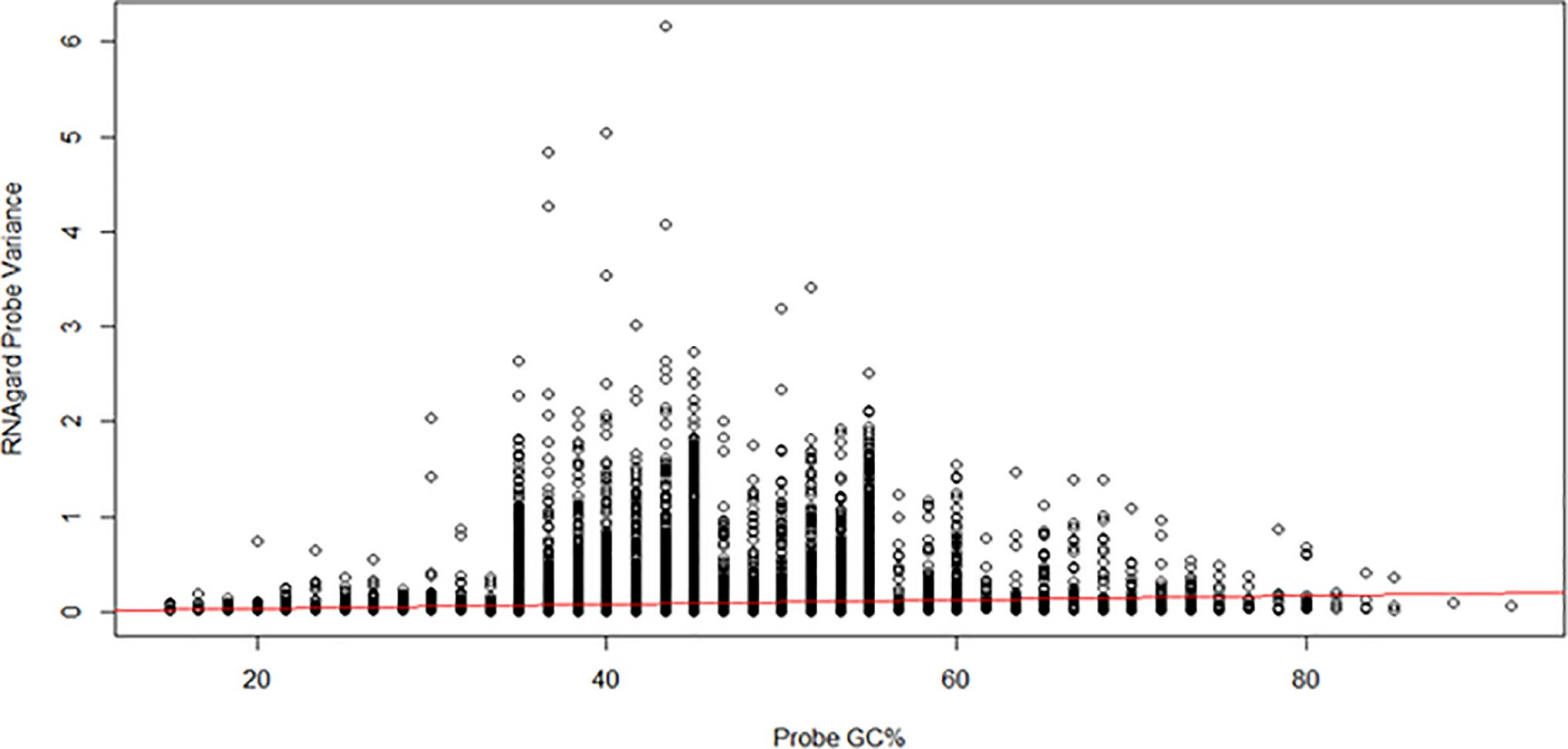
Modest positive trend of increased probe variance and GC content. Scatter plot showing RNAgard^®^ probe variance by GC%. Red line shows linear regression fit.

In studies of RNA degradation, probe positional and sequence properties have been shown to significantly affect measured expression levels. For example, Gallego Romero et al. [3] found that coding DNA sequence (CDS) length and guanine-cytosine (GC) content were both significantly (cor.test function of “stats” R package, p<0.01) positively correlated with degradation rates. Table 2 summarizes correlations observed in each preservation system, in terms of technical variations related to mRNA probe variability and transcript physical properties. It can be seen that overall, the correlation between measures of probe variation and physical properties are small in magnitude. Relatively small correlations between probe error and physical transcript properties suggest differential mRNA degradation is not driving PAXgene^®^RNA/RNAgard^®^ differences. For example, a slightly positive correlation between gene variance and GC percentage is seen among the RNAgard^®^ samples (Fig 3); however, this value is not large enough to account for the differences observed between the collection methods. PBMC expression levels showed larger magnitude positive correlation with 5’ distance, 3’ distance, gene size, and transcript size than PAXgene^®^RNA or RNAgard^®^.

**Table 2:** Correlation of within donor and preservation method probe error with probe properties.

	PAXgene^®^RNA					RNAgard^®^					PBMC/Trizol				
	Var	p-val	FD	vs Gard	vs PBMC	Var	p-val	FD	vs Pax	vs PBMC	Var	p-val	FD	vs pax	vs gard
Dist3	-0.009	0.001	**0.032**	**0.031**	**0.035**	**0.000**	0.006	**0.054**	0.031	**0.056**	**-0.022**	0.008	**0.060**	**0.035**	**0.056**
Dist5	**-0.034**	0.013	0.000	0.001	**0.076**	**-0.026**	**0.024**	**0.027**	0.001	**0.054**	**-0.036**	0.004	**0.031**	**0.076**	**0.054**
TranscriptSize	**-0.034**	0.012	0.009	0.008	**0.081**	**-0.025**	**0.025**	**0.040**	0.008	**0.065**	**-0.040**	0.005	**0.044**	**0.081**	**0.065**
GeneSize	**-0.032**	0.013	0.012	0.003	**0.087**	-0.021	**0.024**	**0.046**	0.003	**0.068**	**-0.036**	0.007	**0.049**	**0.087**	**0.068**
GCpercent	0.017	-0.010	0.021	**0.072**	**-0.056**	**0.088**	-0.011	**0.045**	**0.072**	**-0.035**	-0.001	-0.006	**0.061**	**-0.056**	**-0.035**
meanM	**0.312**	**-0.101**	**0.150**	**-0.258**	**-0.223**	**0.286**	**-0.024**	**0.043**	**-0.258**	**-0.196**	**0.244**	**-0.067**	0.013	**-0.223**	**-0.196**
meanA	-0.007	**-0.171**	**-0.221**	**-0.252**	**-0.384**	-0.020	**-0.159**	**-0.357**	**-0.252**	**-0.398**	**-0.023**	**-0.124**	**-0.477**	**-0.384**	**-0.398**

Within each platform the mRNA probe variability was measured by mean within individual variance (Var), p-value (p-val), and absolute fold difference (FD). Across platforms (vs PAXgene^®^RNA, vs RNAgard^®^, vs PBMC/TRIzol.), p-values were calculated for each probe using a t-test across the same individuals. Numbers provide Pearson’s correlation values between probe variability measures (columns) and probe distance to 5’ and 3’ ends of transcript, gene and transcript size, G+C nucleotide percent, mean probe FD vs. universal reference mRNA (M), and mean probe intensity (A). Bold values represent significant correlation at p<0.01. The data is also represented as the S1 Fig.

When comparing the two whole blood systems, genes from RNAgard^®^ and PAXgene^®^RNA were ordered by their technical variance, where the variance of each gene was measured and averaged across the technical replicates from the same subject. Table 3 shows the top 10 enrichment GO (Gene Ontology) terms for the RNAgard^®^ and PAXgene^®^RNA platforms. In both systems, considerable variations were enriched in genes associated with immune response and homeostasis functions. Other studies have reported differences in the expressions of immune function related genes in response to phytohemagglutinin stimulation using the PAXgene^®^RNA and Tempus^™^ systems, which has important implications in monitoring immune system changes in humans [7].

**Table 3:** Biological processes as indicated by specific Gene Ontology Id’s most highly associated with variable mRNA within RNAgard^®^ and PAXgene^®^RNA preservation methods.

GO Term		
**RNAgard^®^**	Gene Count	FDR
GO:0006955~immune response	52	3.3E-06
GO:0006952~defense response	48	5.2E-06
GO:0009615~response to virus	18	4.0E-05
GO:0006954~inflammatory response	25	7.8E-02
GO:0009617~response to bacterium	18	1.2E-01
GO:0002831~regulation of response to biotic stimulus	7	3.2E-01
GO:0045087~innate immune response	14	4.4E-01
GO:0055066~di-, tri-valent inorganic cation homeostasis	19	5.5E-01
GO:0006916~anti-apoptosis	17	8.2E-01
GO:0030005~cellular di-, tri-valent inorganic cation homeostasis	18	8.4E-01
**PAXgene^®^RNA**		
GO:0006955~immune response	92	1.2E-25
GO:0006952~defense response	71	2.3E-15
GO:0006954~inflammatory response	40	3.8E-08
GO:0009615~response to virus	23	4.6E-08
GO:0009611~response to wounding	50	1.2E-06
GO:0055066~di-, tri-valent inorganic cation homeostasis	28	1.8E-04
GO:0030005~cellular di-, tri-valent inorganic cation homeostasis	27	2.3E-04
GO:0009897~external side of plasma membrane	23	3.1E-04
GO:0048584~positive regulation of response to stimulus	27	5.0E-04
GO:0001775~cell activation	30	6.6E-04

Top 10 enrichment GO terms for genes within each platform with top 5% mean within individual variance using the DAVID annotation tool with gene count and FDR derived from the tool are listed here [11, 17].

Finally, we compared within-subject replicate samples between the PAXgene^®^RNA and RNAgard^®^ systems to identify genes that were differentially affected in these two preservation methods. As shown in Table 4, genes related to nuclear lumen and apoptosis were found to be higher in the RNAgard^®^ system, while metal ion binding genes showed a higher value in the PAXgene^®^RNA platform. Similar differential enrichment of genes associated with intracellular organelles, most notably the nucleus and mitochondria, were identified by GO analysis in blood transcriptomic profiles obtained using three different collection methods [18].

**Table 4:** Biological processesfunctions as indicated by specific Gene Ontology Id’s Gene Ontology terms associated with differentially expressed genes in the RNAgard^®^ and PAXgene^®^RNA systems.

GO Term	Gene Count	FDR
**RNAgard^®^**		
GO:0031981~nuclear lumen	66	1.3E-04
GO:0005829~cytosol	62	1.6E-04
GO:0005654~nucleoplasm	46	5.7E-04
GO:0019899~enzyme binding	33	2.2E-03
GO:0042981~regulation of apoptosis	42	3.9E-03
GO:0043067~regulation of programmed cell death	42	5.1E-03
GO:0010941~regulation of cell death	42	5.5E-03
GO:0070013~intracellular organelle lumen	70	1.0E-02
GO:0043065~positive regulation of apoptosis	27	1.8E-02
GO:0043068~positive regulation of programmed cell death	27	2.0E-02
**PAXgene^®^RNA**		
GO:0008270~zinc ion binding	59	0.01
GO:0046914~transition metal ion binding	65	0.06
GO:0045824~negative regulation of innate immune response	3	2.11
GO:0043087~regulation of GTPase activity	8	3.81
GO:0008380~RNA splicing	12	5.47
GO:0003723~RNA binding	21	5.06
GO:0045793~positive regulation of cell size	5	8.67
GO:0015935~small ribosomal subunit	5	7.84
GO:0043169~cation binding	78	8.79
GO:0046872~metal ion binding	77	10.33

Top 10 enrichment GO terms and FDRs (False Discovery Rate) for those genes which were relatively higher (p<0.05) in the preservation systems tested, using the DAVID annotation tool with gene count and FDR derived from the tool are listed here [11, 17].

## Discussion

Robust detection of transcript levels and high reproducibility in conjunction with minimal experimental variance are critical in discerning true biological differences. While standardization of blood sample handling and processing procedures are essential within experiments, few studies have investigated the influence of sample collection and its impact on whole blood transcriptome analysis. Changes observed in peripheral blood expression profiles due to *ex vivo* alterations in mRNA transcripts call for reliable experimental protocols that assure that data obtained are accurate reflections of the true physiological status of the individuals. To address this discordance, a number of commercial products for blood collection and preservation have recently evolved, and studies evaluating these methods have been reported. We have compared gene expression profiles across technical replicate samples collected in PAXgene^®^RNA and RNAgard^®^, which are two proprietary commercial preservation systems.

Overall, large differences were consistently noted between samples collected from the same individual when these two different systems were used. These variations persisted even after both within and between microarray normalizations were applied. These observations strongly caution against direct comparison of results from samples preserved in RNAgard^®^ with those preserved in PAXgene^®^RNA, as they may give rise to methodologically related false positive results.

Similarly, when results must be compared between diverse methods of preservation, particularly if differences are noted in specific gene types and their function, additional care should be exercised in the interpretation. Although the order of gene variances did not change much significantly between the PAXgene^®^RNA and RNAgard^®^ platforms, it is possible that increased noise or signal reduction related to specific components of the preservation system could mask expression changes under some experimental conditions. We would like to emphasize that the gene specific change in signal/noise is much harder to correct than may other types of batch-to-batch differences, such as hybridization efficiency or image quality, that generally have a linear component(s) that can be identified and, at least partially removed. Our attempt to identify links between technical variability and the probe properties pointed to some possible contributors affecting differential expression. Also, an examination of the enrichment of annotation terms indicated considerable differences in genes related to specific functions. The RNAgard^®^ system showed remarkably high numbers for apoptosis associated genes, while the PAXgene^®^RNA product showed mostly high numbers for enriched genes related to metal ion binding. It is possible that variations in the rate of cell lysis between the two methods may explain the observed differential expression of apoptosis genes, while inclusion of chelators may account for the observed differences in metal ion binding gene expression.

Despite differences in cellular makeup, previous studies have shown general comparability when looking at PBMCs vs whole blood gene expression [19]. The lower signal-to-noise ratios seen in this study may reflect experimental design more than the underlying stability of the systems. Here, due to volume requirements, two CPT tubes were collected per person vs one each for PAXgene^®^RNA and RNAgard^®^. Additionally, PBMC separation presents an additional processing step. Both of these differences are likely to add technical variation. In this case, any potential gains from having “purified” cell types and higher RIN values did not compensate for this increased variation. Due to these underlying cellular and methodological differences, this study focused on comparing PAXgene^®^RNA and RNAgard^®^ with PBMCs in Trizol to provide a larger frame of reference.

In conclusion, our study compared the PAXgene^®^RNA and RNAgard^®^ systems for the first time, and demonstrated distinct differences between the whole blood gene expression profiles generated using these two collection platforms. By comparing gene expression measurements within and across technical replicates from the same individuals, we found systematic differences between PAXgene^®^RNA and RNAgard^®^. Minimal signal-to-noise differences were observed in the samples in the study. The results of these studies strongly suggests the deployment of a single preservation platform throughout the course of any specific study without mixing or changing of the collection method during the course of sample acquisition and preservation.

## Supporting information

S1 Fig Scatterplot: Scatterplot representing correlation between donors and preservation methods (A) PAXgene^®^ vs PBMCs (B) RNAgard^®^ vs PAXgene^®^ (C) PBMCs vs RNAgard^®^.
(TIF)
