# Supplementary figures and images for: Gene expression profiling of whole blood: A comparative assessment of RNA-stabilizing collection methods
Source: PLoS One. 2019 Oct 10;14(10):e0223065. doi: 10.1371/journal.pone.0223065 (PMC6786555; doi:10.1371/journal.pone.0223065)

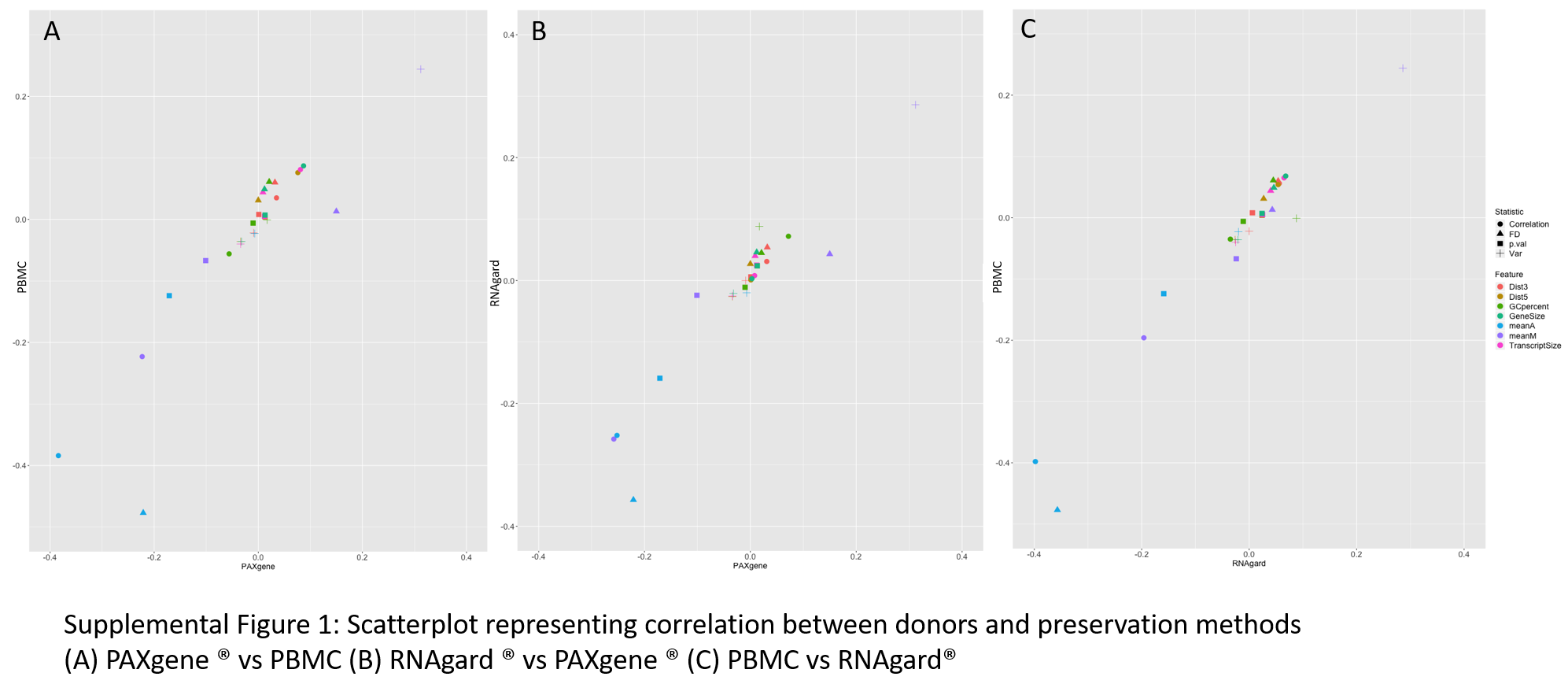

Supplement: S1 Fig — Scatterplot: Scatterplot representing correlation between donors and preservation methods (A) PAXgene® vs PBMCs (B) RNAgard® vs PAXgene® (C) PBMCs vs RNAgard®. (TIF) [file pone.0223065.s001.tif]
